# Synergistic Impact of the Symptom Cluster on Health-Related Quality of Life in Patients With Chronic Obstructive Pulmonary Disease: A Secondary Data Analysis

**DOI:** 10.1177/10547738221085765

**Published:** 2022-04-01

**Authors:** Fei Fei, Jonathan Koffman, Xiaohan Zhang, Wei Gao

**Affiliations:** 1 King’s College London, UK; 2 Jiangsu College of Nursing, Huai’an, P.R. China

**Keywords:** chronic obstructive pulmonary disease, health-related quality of life, secondary analysis, symptom clusters, symptom management

## Abstract

This study aimed to quantify the synergistic impact of symptom clusters on health-related quality of life (HRQoL) among patients with chronic obstructive pulmonary disease (COPD). We conducted a secondary analysis of a cross-sectional data collected via convenience sampling from patients with COPD. Multiple linear regressions were used to quantify the relationships between symptom clusters and HRQoL. The sample included 106 COPD patients from whom three symptom clusters were identified comprising of dyspnea depression, anxiety-sleep, and depression-anxiety. Depression-anxiety (psychological symptom cluster) was significantly associated with poorer HRQoL (β = 13.88, 95% CI [7.94, 19.82]), while no significant associations were detected with HRQoL for either depression or anxiety alone (β = 6.66, 95% CI [−2.99, 16.31]; β = 7.29, 95% CI [−0.78, 15.35]). Assessment and early intervention led by nurses targeting the psychological symptom cluster may represent an initial approach to improve HRQoL. Understanding the phenomenon of symptom clusters that are present in patients with COPD provides a breakthrough insight to devise strategies for their management.

## Introduction

Chronic obstructive pulmonary disease (COPD) is one of the leading causes of morbidity worldwide and is anticipated to become the third leading cause of death globally by 2030 (Global Initiative for Chronic Obstructive Lung Disease, 2022). Previous studies have investigated the effectiveness of different management strategies for single symptoms associated with COPD; however, a large proportion of people with this disease experience multiple concurrent symptoms (Jenkins et al., 2019). This phenomenon corresponds with what is referred to as a “symptom cluster,” which implies a group of two or more symptoms that occur together and are related to each other (Blakeman, 2019). Symptom management science spotlights a conversion from focusing on targeting single symptoms to symptom clusters that may more efficiently improve clinical symptom management (Fei, Siegert, et al., 2022). Although increasing evidence has shown that symptom clusters occur in patients with COPD, there is no consensus on the definition of symptom clusters either methodologically or conceptually as well as in clinical practice and research (Miaskowski et al., 2017). Validation of the concept of symptom cluster is complicated and difficult because evaluating symptom inter-relationships manifests a host of methodological challenges, specifically the study design, theoretical frameworks, measurement tools, symptom dimensions, statistical methods to identify clusters, statistical “cut-off” points to define clusters and characteristics of study samples may all contribute to these inconsistencies of symptom clusters (Xiao, 2010).

Dyspnea is the most frequently experienced symptom associated with COPD and a major cause of anxiety and depression (Global Initiative for Chronic Obstructive Lung Disease, 2022). Specifically, it is described as a sense of increased effort to breathe, chest heaviness, air hunger, or gasping (Vogelmeier et al., 2020). Anxiety and depression are also common in COPD, especially in the severe stage of the disease (Global Initiative for Chronic Obstructive Lung Disease, 2022), which has been identified as being associated with poorer health status and increased risk of exacerbations (Blakemore et al., 2019). Research has also identified the prevalence of sleep disorders can be present in up to 48% of patients with COPD (Ohayon, 2014), where anxiety and depression may account for the etiology of sleep disorders (Budhiraja et al., 2015). It appears that these symptoms may cluster together or in some instances, reciprocally influence each other.

Different approaches have been proposed to identify the presence of symptom clusters. For example, the “Most-Common Symptom Approach,” summarized in a systematic review, is known as “a priori” cluster identification where researchers initially selected several commonly experienced symptoms assuming that the most common symptoms could be grouped together as “clusters” (Xiao, 2010). The cluster identification statistical analysis used most frequently in this approach is clustering by correlations between symptoms (Xiao, 2010). Compared with the “most common symptom approach,” the “all-possible symptom approach,” targets all potential symptoms that patients with COPD might experience when identifying clusters (Xiao, 2010). The cluster identification statistical analysis used most frequently in this approach is exploratory factor analysis (EFA; Fei, Koffman, et al., 2022). Our recently published systematic review (Fei, Koffman, et al., 2022) states that researchers must be clear about what kind of approaches should be adopted for symptom cluster identification and to fit their study objectives. In this study, we adopted the “Most-Common Symptom Approach” of symptom cluster identification aiming to fit our study objectives.

The “Theory of Unpleasant Symptoms” (TOUS) provides a comprehensive structure to understand symptoms and represents a middle-range theory that has direct application to clinical practice (Blakeman, 2019). Moreover, theories may help inform practice, while practice may also inform the further development of theories (Smith & Liehr, 2014). Based on clinical experiences, the TOUS assumes that when compared with singular symptoms, the presence of symptom clusters may significantly increase symptom burden and synergistically influence patients’ health-related quality of life (HRQoL; Lenz et al., 1997). However, to date, no studies have robustly examined the synergistic impact of the symptom cluster associated with COPD on HRQoL in the COPD population. This study addressed this research gap and aimed to quantify the synergistic impact of the symptom clusters associated with COPD on HRQoL in the COPD population compared with singular symptoms.

## Methods

### Design

This study represents a secondary data analysis of a cross-sectional survey via convenience sampling designed to examine factors associated with HRQoL in patients with COPD. The study was approved by the university institutional review board (approval number: HR-18/19-13608) and the hospital ethical committee (approval number: HEYLL20181A).

### Setting and Sample

This cross-sectional study was conducted between January to August 2018 at the Department of Respiratory and Critical Care Medicine within tertiary health care in Huai’an city, Jiangsu province, China. This study is reported following STROBE guidelines for reporting of observational (Von Elm et al., 2014). The eligible criteria for patients to be included in the secondary data analysis were as follows: (i) a diagnosis of COPD as confirmed by a pulmonologist according to the post-bronchodilator Spirometry, (ii) the forced expiratory volume in 1 second (FEV1)/Forced vital capacity (FVC) ≤70% on pulmonary function test, (iii) those aged 18 years or older, and (iv) the ability to provide written informed consent. Patients who had speech or hearing difficulties and cognitive disorders were excluded. A total of 106 participants was investigated. Post hoc power analysis using G-Power 3.1.7 indicated that 106 study participants yielded 99.9% power with an effect size (*f*^2^) of 0.53, calculated by a formula using multiple *R*^2^ at a significance level of .05 (two-sided; Verma & Verma, 2020).

### Data Collection and Measurements

Clinicians identified eligible patients by screening medical records. Eligible participants were fully informed about the purpose of the study, questionnaires, and time commitments and its potential benefits and risks. Researchers subsequently conduct a face-to-face interview to elicit the presence of symptoms and measure their HRQoL.

The socio-demographic and clinical characteristics of participants were assessed using a self-completed socio-demographic and clinical questionnaire. Dyspnea was measured using the modified Medical Research Council (mMRC) Dyspnea Scale, which consists of one item with five statements that describe the entire range of dyspnea from none (Grade 0) to being completely incapacitated by the illness (Grade 4) with a clinical cut-off point of two (Launois et al., 2012). There are no other reports of the reliability and validity of this measure at the time of its development (Eakin et al., 1993). However, researchers have subsequently attempted to test the validity of the mMRC Dyspnea Scale by examining its relationship with the other measures of health status, for example, the ability to walk a pre-defined distance (Camargo & Pereira, 2010) or with the COPD Assessment Test (Rhee et al., 2015), all of which indicate that this scale is valid and correlates with clinical parameters and parameters of respiratory function in COPD patients (Global Initiative for Chronic Obstructive Lung Disease, 2022).

Depression was measured using the 4-point 21-item Beck Depression Inventory (BDI; Beck et al., 1961). Based on the severity of the symptom, the total score ranges from 0 to 63, where scores from 0 to 9 indicate “no or minimal depression”; scores from 10 to 18 indicate “mild depression”; scores from 19 to 29 indicate “moderate depression”; and scores from 30 to 63 indicate “severe depression” (Beck et al., 1961). Good internal consistency (Cronbach’s α = .94) of BDI was reported in the Chinese population (Wang et al., 2011). In this study, the Cronbach’s alpha of BDI was .87, and a cut-off point of 9 was used to distinguish participants being depressed and not depressed.

Anxiety was measured using the 4-point 21-item Beck Anxiety Inventory (BAI; Beck et al., 1988). Based on the severity of symptoms, the total score ranges from 0 to 63, where scores from 0 to 7 indicate “no or minimal anxiety”; scores from 8 to 15 indicate “mild anxiety”; scores from 16 to 25 indicate “moderate anxiety”; and scores from 26 to 63 indicate “severe anxiety” (Beck et al., 1988). Satisfactory reliability (Cronbach’s α = .95) and validity of BAI were reported in the Chinese population (Pang et al., 2019). In this study, the Cronbach’s alpha of BAI was .91, and a cut-off point of 7 was used to distinguish participants being anxious and not anxious.

Sleep quality was measured using a self-rated 19-item Pittsburgh Sleep Quality Index (PSQI; Buysse et al., 1989). The 19 items are combined into seven components with scores from 0 to 3. The 7 component scores are added to produce a global score ranging from 0 to 21, with higher scores indicating worse sleep quality and a cut-off point of 5 distinguishing “good” and “poor” sleepers (Buysse et al., 1989). The Chinese version of the PSQI has been shown to have acceptable internal consistency (Cronbach’s alpha = .71; Ho et al., 2021). In this study, the Cronbach’s alpha of PSQI was .77.

HRQoL was measured using the St George’s Respiratory Questionnaire (SGRQ; Jones et al., 1991). SGRQ is a self-administered, 50-items scale with three dimensions: symptoms, activity, and impact on daily life (Jones et al., 1991). The total scores of SGRQ range from 0 to 100, where 0 indicates no impairment on quality of life and higher scores represent worse HRQoL. The SGRQ was reliable in a study conducted in a Chinese population with good internal consistency (Cronbach’s α = .87; Xu et al., 2009). In this study, the Cronbach’s alpha of SGRQ was .80.

### Data Analysis

Descriptive statistics were used to summarize the patients’ sociodemographic and clinical characteristics. According to the cut-off for each of the scales used to measure symptoms, we transformed symptom-related variables into binary variables (0 = without a symptom, 1 = with a symptom). As proposed by Xiao (2010), chi-squared tests, odds ratio (OR), and associated 95% confidence intervals (95% CI) were calculated to test the association between symptoms for identification of symptom clusters. Multiple linear regression (MLR) was used to examine the impact of symptom clusters on HRQoL compared with singular symptoms. To justify the sample size, the standard that 10 cases per predictor variable would produce a valid model of fit was adopted (Peduzzi et al., 1996). Specifically, in a regression model, the total score of HRQoL was calculated and used as a dependent variable. The symptom cluster was represented as a binary variable and modeled as an independent variable. Taking the anxiety-depression cluster as an example, the cluster was assigned the value of 1 if anxiety = 1 and depression = 1. Otherwise, the cluster was assigned the value of zero. Singular symptoms of the same cluster were represented as binary variables and modeled as independent variables. Taking singular anxiety of the anxiety-depression cluster as an example, the variable of singular anxiety was assigned the value of 1 if anxiety = 1 and depression = 0. Otherwise, the variable was assigned the value of zero. Sociodemographic/clinical variables were selected as controlled variables by using Kruskal-Wallis H test used to identify if they had relationships with HRQoL, since HRQoL is a continuous variable with unnormal distribution data (Weaver et al., 2017). All categorical variables were converted into dummy variables while being calculated in MLR. MLR results were reported in terms of beta values (β) and 95% CIs. STATA/IC 15.1 was used for statistical analysis.

## Results

### Sample Characteristics

A total of 106 participants completed the survey, of whom 82 (77.4%) were male, and the largest age group was 71 to 80 years old (*n* = 48; 45.3%). The demographic and clinical characteristics of patients are summarized in Table 1.

**Table 1.** Relationship Between Socio-Demographic/Clinical Variables and Health-Related Quality of Life (*N* = 106).

Variables	Frequency	%	Health-related quality of life	
			χ^2^	*p*-value
Sex				
Male	82	77.4	8.71	<.01
Female	24	22.6		
Age in years				
<60	4	3.8	1.08	.78
60–70	27	25.5		
71–80	48	45.3		
>80	27	25.5		
Living place				
City	56	52.8	0.17	.68
Rural	50	47.2		
Marital status				
Married	95	89.6	0.61	.43
Single	11	10.4		
Living state				
Alone	10	9.4	8.03	.04
Living with spouse	60	56.6		
Living with children	29	26.4		
Living with parents	7	6.6		
Education level				
Elementary school or less	63	59.4	6.08	.11
Middle school	27	25.5		
High school	9	8.5		
≥College	7	6.6		
Hospitalization cost				
Publicly funded medical care	47	44.3	0.96	.62
Commercial insurance	56	52.8		
Self-funded	3	2.8		
Monthly income per Yuan				
<500	30	28.3	6.90	.08
500–1,000<	12	11.3		
1,000–2,000	15	14.2		
>2,000	49	46.2		
Smoking history				
Current smoker	26	24.5	1.70	.43
Never smoker	27	25.5		
Former smoker	53	50.0		
Alcohol history				
Current drinking	18	17.0	3.08	.21
Never drinking	42	39.6		
Former drinking	46	43.4		
Exercise situation				
Never	51	48.1	5.32	.15
≤2 times per week	35	33.0		
3–5 times per week	14	13.2		
>5 times per week	6	5.7		
Time since diagnosed (years)				
1–10	54	50.9	0.66	.88
11–20	27	25.5		
21–30	11	10.4		
>30	14	13.2		
Stage of COPD				
Gold I (mild)	2	1.9	17.69	<.01
Gold II (moderate)	37	34.9		
Gold III (severe)	53	50.0		
Gold IV (very severe)	14	13.2		
Number of exacerbations in the previous 12 months				
No	6	5.7	9.00	.03
One	32	30.2		
Two	38	35.9		
≥Three	30	28.3		
Days in hospital in the previous 12 months				
≤14	37	34.9	2.00	.57
15–28	54	50.9		
29–42	8	7.6		
≥42	7	6.6		

*Source*. Global Initiative for Chronic Obstructive Lung Disease (2022).

*Note*. Gold = global initiative for chronic obstructive lung disease.

### Symptom Clusters in Patients With COPD

Three symptom clusters were identified among patients with COPD (Table 2): dyspnea-depression cluster (OR = 2.69, 95% CI [1.19, 6.02]), anxiety-sleep cluster (OR = 2.72, 95% CI [1.20, 6.15]), and depression-anxiety cluster (OR = 6.13, 95% CI [2.57, 14.60]).

**Table 2.** Symptom Clusters in COPD Patients (*N* = 106).

Dyspnea	Depression		Total	OR	95% CI
	Yes	No			
Yes	24	25	49	2.69	[1.19, 6.02]
No	15	42	57		
Total	39	67	106		
Depression	Anxiety		Total	OR	95% CI
	Yes	No			
Yes	27	12	39	6.13	[2.57, 14.60]
No	18	49	67		
Total	45	61	106		
Anxiety	Sleep		Total	OR	95% CI
	Yes	No			
Yes	32	13	45	2.72	[1.20, 6.15]
No	29	32	61		
Total	61	45	106		
Dyspnea	Anxiety		Total	OR	95% CI
	Yes	No			
Yes	24	25	49	1.65	[0.76, 3.58]
No	21	36	57		
Total	45	61	106		
Dyspnea	Sleep		Total	OR	95% CI
	Yes	No			
Yes	34	15	49	2.52	[0.97, 5.60]
No	27	30	57		
Total	61	45	106		
Depression	Sleep		Total	OR	95%CI
	Yes	No			
Yes	26	13	39	1.83	[0.81, 4.15]
No	35	32	66		
Total	61	45	106		

### Associations Between Symptom Clusters and HRQoL

Patient gender, living state, stage of disease, and number of COPD exacerbations in the last 12 months were selected as covariates to be added into the multiple linear regression (Table 1). According to the multiple linear regression adjusted for the covariates above, all three symptom clusters (anxiety-sleep, dyspnea-depression, and depression-anxiety) were significantly associated with poorer HRQoL (β = 14.88, 95% CI [7.19, 22.56]; β = 14.21, 95% CI [6.08, 22.33]; β = 13.88, 95% CI [7.94, 19.82]; Table 3). Furthermore, with regards to associations between symptom clusters/singular symptoms and HRQoL, we identified that the depression-anxiety cluster was significantly associated with poorer HRQoL (β = 13.88, 95% CI [7.94, 19.82]). However, singular depression symptom (β = 6.66, 95% CI [−2.99, 16.31]) and anxiety symptom (β = 7.29, 95% CI [−0.78, 15.35]) were not associated with HRQoL.

**Table 3.** The Association Between Symptom Clusters and Health-Related Quality of Life (*N* = 106).

Variables	Health-related quality of life	
	Coefficient	95% CI
Anxiety and sleep cluster	14.88	[7.19, 22.56]
Anxiety	10.09	[−0.40, 20.59]
Sleep	7.41	[−1.66, 16.48]
Dyspnea and depression cluster	14.21	[6.08, 22.33]
Dyspnea	8.18	[0.49, 15.88]
Depression	11.67	[1.76, 21.58]
Depression and anxiety cluster	13.88	[7.94, 19.82]
Depression	6.66	[−2.99, 16.31]
Anxiety	7.29	[−0.78, 15.35]

## Discussion

### Main Findings

This is the first study to examine the synergistic impacts of symptom clusters on HRQoL when compared to the presence of singular symptoms in patients with COPD. Our findings empirically supported the “Theory of Unpleasant Symptoms” that assumed symptoms in a cluster were shown to exert synergistic effects on HRQoL (Lenz et al., 1997). We also identified that the psychological symptom cluster (anxiety-depression cluster) had a synergistic impact on HRQoL. Multiple linear regression represents a statistical technique for evaluating the relationship among variables. The main advantage of multiple linear regression is the ability to determine the relative influence of predictor variables on the criterion value (Olive, 2017). The beta coefficient is the degree of change in the outcome variable in multiple linear regression. The higher the absolute value of the beta coefficient, the stronger the association (Alexopoulos, 2010). Specifically, based on findings from this study, the beta value of the psychological symptom cluster was higher than that of the depression symptom or the anxiety symptom alone. This implies the association between the psychological symptom cluster and HRQoL we identified is stronger than the association between singular symptoms and HRQoL.

Previous studies (Jang et al., 2019; Montserrat-Capdevila et al., 2017) have identified that anxiety and/or depression influence HRQoL in the COPD patient population. However, there is a possibility that these studies did not exclude participants with prior or concurrent anxiety and depression symptoms. Although the mechanism of the psychological symptom cluster’s synergistic impact on HRQoL remains obscure, Jacobson and Newman (2017) reported that anxiety and depression might represent a bi-directional risk factor for one another. This means that patients with COPD who experience concurrent anxiety and depression may also experience severe psychological symptom burden leading to a commensurate decrease in their HRQoL.

### Symptom Clusters in COPD Patients

Our study identified three symptom clusters in patients with COPD: dyspnea-depression, depression-anxiety, and anxiety-sleep. Similar to our finding of the dyspnea-depression cluster, Kunik et al. (2005) identified that depression often occurred together with breathing problems. A strong association between psychological symptoms and dyspnea was also found in prior research (Schuler et al., 2018). In addition, the etiology and mechanism of the increased prevalence of depression in patients with COPD are not well understood (Jang et al., 2019).

COPD causes irreversible airflow limitation resulting in a decrease in the oxygen supply to the brain and loss of regional grey matter accompanied by impairment of white matter microstructural integrity which is associated with disease severity and might account for the psychological changes of COPD (Zhang et al., 2012). Screening for the presence and levels of depression may be necessary for people with COPD. The anxiety-sleep cluster has not been observed in previous studies. The lack of congruence between the findings from our study and prior research might be due to differences in symptom measurements and the statistical methods employed among the studies (Jenkins et al., 2019). However, some studies reported anxiety had been shown to have a negative effect on sleep quality in the COPD population (Budhiraja et al., 2015). This finding highlighted when anxiety or sleep disorder was identified, nurses might anticipate, and probe further into the other related symptom (Aktas et al., 2010). The depression-anxiety cluster was consistent with the study, which identified the mood cluster consisted of depression and anxiety in South Korean patients with COPD (Lim et al., 2017).

Our study identified that all the symptom clusters were significantly associated with poorer HRQoL in the COPD patient population. Moreover, the relationship between the depression-anxiety cluster and HRQoL identified in this study is supported by findings from previous research that observed that the psychological symptom cluster was a significant factor associated with poor HRQoL (Wu et al., 2020). Therefore, our results reinforce the significant effect of the psychological symptom cluster on HRQoL in people living with COPD. The other two symptom clusters influencing HRQoL were the dyspnea-depression and the anxiety-sleep cluster. Although no prior studies have assessed the correlation between these two symptom clusters and HRQoL in patients with COPD, our results e corresponded with earlier research on individual symptoms observed with this patient group (Yohannes & Alexopoulos, 2014). These findings should be considered when developing targeted and effective symptom management strategies to improve HRQoL in patients with COPD. Specifically, symptom management programs should pay more attention to the presence of a psychological symptom cluster as this may lead to considerable distress associated with poor HRQoL.

### Limitations

This study has a number of limitations that influence the inferences that can be taken from the findings presented. First, the sample size in this study was relatively small, which may lead to type II error associated with a decrease in the likelihood of detecting a significant difference when one truly exists. Second, this study did not record any data on the type of comorbidities. Therefore, it may be difficult to clarify symptoms that are attributable to COPD. Third, information concerning anxiety and depression may have been underestimated due to Chinese culture, where these issues are often not verbalized (De Vaus et al., 2018). Specifically, psychologists have reported that patients from Chinese cultures may be more likely to report and focus on physical symptoms at the expense of psychological symptoms of anxiety and depression, which may nevertheless still be present, than in Western countries. Finally, the findings were limited in terms of their general applicability since data collection was limited to one university hospital and four symptoms of the participants; therefore, large-scale studies focusing on more COPD symptoms are needed to understand COPD symptom clusters.

### Relevance to Clinical Practice

Our study identified that patients with COPD experience co-existing anxiety and depression symptoms, which are frequently grouped as a cluster with synergistic impact on HRQoL. Nurses caring for patients with COPD should pay more attention to the nature of the interactions among symptoms within a cluster rather than focusing on singular symptoms. Furthermore, it is important that when nurses routinely assess patients with COPD, they specifically lookout for the presence of concurrent anxiety and depression symptoms, which have been demonstrated as synergistically impacting HRQoL. Moreover, symptom management strategies capable of collectively managing psychological symptoms (e.g., cognitive behavioral techniques) may offer utility in effectively improving COPD-associated HRQoL. Future research is required to further investigate the presence of concurrent symptom management strategies tailored to patients with COPD and develop strategies to mitigate distress where it is present.

## Conclusion

This study provides important new evidence on the science of examining the presence of clinically significant symptom clusters among patients with COPD. The present study provides an emerging empirical basis for focusing on symptom clusters and associated HRQoL outcomes. To our knowledge, this is the first study to observe the synergistic impact of the psychological symptom cluster on HRQoL in patients with COPD as compared with single symptoms. Given the findings in this study, we believe incorporating impeccable assessment of the potential presence of symptom clusters in this patient population may facilitate the earlier identification. Failure to do so may have implications for the subsequent treatment of symptoms that taken together have an additive and detrimental effect on HRQoL. This may provide a better understanding of this phenomenon which can contribute to efficient symptom management and alleviate symptom distress among patients with COPD.
